# Prevalence of Allergies among University Students: A Study from Ajman, United Arab Emirates

**DOI:** 10.1155/2014/502052

**Published:** 2014-02-19

**Authors:** Lisha Jenny John, Sharfaa Ahmed, Fiza Anjum, Mohieddin Kebab, Naik Mohammed, Haitham Darwich, Nusaibah Ibraheem, Mohamed Arifulla, Jayadevan Sreedharan

**Affiliations:** ^1^Department of Pharmacology, Gulf Medical University, Ajman 4184, UAE; ^2^College of Medicine, Gulf Medical University, Ajman 4184, UAE; ^3^Statistical Support Facility, CABRI, Gulf Medical University, Ajman 4184, UAE

## Abstract

*Aim*. Urbanization and globalization in the Middle East have resulted in drastic environmental changes and increased allergens present in the environment. This study aimed to assess the prevalence of allergies among undergraduate students from a university. *Material and Methods*. This cross-sectional survey was carried out among undergraduate students of a University at Ajman, UAE. A self-administered questionnaire was used as research instrument for data collection. The demographic data and the allergy characteristics were collected and analyzed using SPSS version 19. Descriptive and inferential statistics were performed. *Results*. A total of 255 students (33.3% males; 66.7% females) were included. Commonest allergies among the students were allergic conjunctivitis (104 (40.8%)), allergic dermatitis (89 (34.9%)), and eczema (38 (14.9%)). Family history of allergies was strongly associated with occurrence of allergic conjunctivitis and allergic dermatitis. In about 58 (22%) of the students, dust was the most common triggering factor for allergies. Allergies associated with pollen, food, and drugs were less frequent. The distribution of allergies based on gender revealed female preponderance in all types of allergies. Students with allergies reported interference with their daily activities, and academic, social, and extracurricular activities. *Conclusions*. Allergic conjunctivitis and allergic dermatitis were the frequent allergies reported. Adequate preventive strategies can crumb the prevalence of allergies.

## 1. Introduction 

Atopic diseases have a wide range of manifestations across the globe, imposing heavy social and economic burden due to their chronicity and effects on various functions of the body [1]. Allergies during college years impact the quality of life by interfering with the daily activities, poor attendance to college, sleep disturbances, and inability to perform academical as well as extracurricular activities. Studies have also documented that allergies are more common in the urban and industrialized societies than the less industrialized regions [2]. Von Hertzen and Haahtela from Finland, reported an increase in the occurrence of allergies among individuals living in the city [3]. Previous studies carried out in the UAE revealed a prevalence rate of 7.3% of asthma and allergic rhinitis and were caused mainly by pollen, mould spores, and dust mite allergens in the air. Prevalence of food allergy among children in UAE is 8% with a significant association with family history [4].

The risk factors attributed to the increasing prevalence of allergies among the Gulf countries include globalization resulting in substantial environmental changes such as increased exposure to pollen due to antidesertification projects and genetic factors may also contribute to the prevalence in certain ethnic groups [5]. Epidemiological studies from the Gulf region on allergic disorders have shown significant discrepancies in the prevalence of asthma and other allergic disorders as well as variations in the possible risk factors [6–8].

On determining the prevalence of allergies among students and any association between gender, ethnic groups and length of stay in the region with the allergies would provide useful information to suggest strategies among students to modification and suggestions to improve their health. Hence, this study aimed to assess the prevalence of allergies among undergraduate students of a University at Ajman, UAE.

## 2. Material and Methods 

This cross-sectional survey was carried out over a period of 6 months (September 2011–February 2012) among undergraduate students of the four colleges of the University. Approval for the study was obtained from the Ethics Committee for conducting this study. All students willing to participate in the study were included. Students who were not willing to participate and those not present during the administration of questionnaire were excluded from the study. Data collection was performed using a self-administered pilot-tested questionnaire with the following domains: sociodemographic characteristics (age, gender, nationality, and length of stay in UAE), allergy characteristics, and family history of allergies. Copies of the questionnaire were handed out to the students during their break.

Descriptive analysis was performed on the data collected using SPSS version 19. Chi- square test was used to test for any association between allergies and variables such as age, gender, nationality, length of stay, and quality of life.

## 3. Results 

A total of 255 students (85 (33.3%) males and 170 (66.7%) females) were included. The mean age was 20 ± 2.6 years. The mean duration of stay in the UAE was about 104.9 ± 90.4 months; the frequency varies from one month to 312 months. The characteristics of the study population are shown in Table 1.

**Figure 1:**
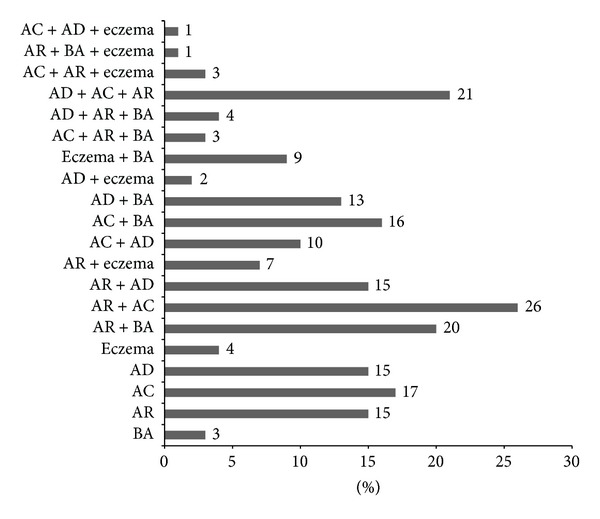
Distribution of allergic disorders among the university students. BA: bronchial asthma; AR: allergic rhinitis; AC: allergic conjunctivitis; AD: allergic dermatitis.

The common allergies self-reported by the students included allergic conjunctivitis 104 (40.8%), allergic dermatitis 89 (34.9%), and eczema 38 (14.9%). The self-reported allergic disorders (single and combination) among the students have been illustrated in Figure 1. Only 54 (21.1%) students reported single allergic disorders. Allergic conjunctivitis and rhinitis were coexistent in 53 students (20.7%) and combination of allergic dermatitis and rhinitis was coexistent in 40 (15.7%) students. Among those students with a family history of allergic dermatitis, 35 (56.5%) of them had allergic dermatitis (*P* < 0.001); those students were reporting positive family history of allergic conjunctivitis and 22 (68.8%) of them had allergic conjunctivitis (*P* < 0.001). Most of the students noted that their allergies were common during spring season of the year (March-May).

The details of gender-based distribution of allergies are detailed in Table 2. A female preponderance was observed in all the types of allergies. There was no association between the type of allergies and length of stay in the UAE or between the allergies and the nationality of students.

Interference with their daily activities, academic activities, and social and extracurricular activities was reported by students with allergies. Allergic conjunctivitis interfered with daily activities, social and extracurricular activities, academic performance, and college attendance in 32 (45.1%), 25 (35.7%), 13 (19.4%), and 11 (16.9%) students, respectively. Allergic dermatitis interfered with daily activities in social and extracurricular activities, academic performance, and college attendance in 28 (46.7%), 9 (29.8%), 11 (19.6%), and 7 (13.5%) students correspondingly. Allergic conjunctivitis and allergic dermatitis and their interference with the daily activities were found to be statistically significant (*P* < 0.01).

Dust was the most common trigger for all the allergies (58 (22%)) (allergic rhinitis, conjunctivitis, dermatitis, and eczema) among the students. The most common food allergies were associated with sea food and nuts. Drug allergies were reported by 18 (7%) students, most frequently to antibiotics, especially the penicillin group of drugs.

## 4. Discussion

United Arab Emirates, as a country, has been through a major transition shifting from traditional to a modern society with urbanization, industrialization, and associated environmental changes resulting in an increase in the allergens present in the environment and the prevalence of allergies. In our study sample, the female students were predominant, with a majority of the students being below 20 years of age.

Dust was the most common trigger for all the allergies (allergic rhinitis, conjunctivitis dermatitis, and eczema). These results indicate that dust plays an important role in worsening allergic symptoms, as has been reported earlier by Bener et al. [9]. Urbanization has changed the home settings immensely with the introduction of soft furnishings, fitted carpets, central cooling, and decreased indoor ventilation which lead to a considerable increase in indoor pollutants and airborne allergens. As noted in this study the allergies were common during spring and the probable trigger is being pollen and dust mites. Similar observation was noted by another study from the United Arab Emirates [10]. Health education should be imparted among the general population regarding the simple measures of reducing dust mites such as washing bedding and nightclothes in hot water, encasing mattress and pillow in dust mite-proof covers, using washable blinds or curtains, and regularly checking air conditioning units for contamination and pest control [11].

The prevalence of allergic conjunctivitis, allergic dermatitis, and eczema were 40.8%, 34.9%, and 14.9%, respectively. The majority of the students had multiple coexistent allergies, allergic conjunctivitis coexistent with rhinitis being the most prevalent. The prevalence of allergic rhinoconjunctivitis in the present study was about 21%; another study from Bangkok reported a prevalence rate of 26% for allergic rhinoconjunctivitis [12]. The prevalence of eczema among undergraduate students was 15%, in comparison to 9.4% reported in the study from Bangkok, and 12.8% from Lebanon [12, 13]. The highest prevalence rate of cutaneous allergy reported from the Middle East was 35.8% in Tehran [14].

Shahar and Lorber from Israel reported prevalence rates of 7%, 6%, and 7% for food allergy, drug allergy, and skin allergy, respectively [15]. In the present study the commonest food allergy was to sea food (crab) followed by nuts, while Al-Hammadi et al. from Al-Ain, UAE, had reported eggs, fruits, and fish as the main allergies in their study [4]. A report from Taiwan documented that protein-rich and fat-rich foods of animal origin were associated with a higher prevalence of allergies among the teenagers [16]. The most frequently reported drug allergies were to antibiotics, particularly to penicillin group. Allergic drug reactions have been reported to cause the highest number of documented deaths from anaphylaxis each year [17]. Hence it is critical for individuals with allergies to identify them and to avoid foods and drugs that may cause allergic reactions.

Family history of allergies was strongly associated with occurrence of allergic conjunctivitis and allergic dermatitis supporting previous publications on allergies from the United Arab Emirates [5, 10]. This finding may be suggestive of a probable genetic influence on the development of allergies among the students. However in the present study we did not observe any association between the allergies and nationality of the student.

The effect of allergies on an individual's quality of life and the extent to which it may restrict daily activities are often ignored. Our study revealed that the allergies had restricted the daily activities of the students. Early identification of the allergens and avoiding them is the primary measure to reduce the occurrence of allergies. Educating and creating awareness regarding the allergic and respiratory diseases especially among the school students would help them identify the allergens and adopt precautionary measures.

Strength of the study is the high response rate of the students, inclusion of adolescents unlike children in most of the studies. The limitation of the study includes nongeneralizability of the findings to the entire population in the region due to the sample size in the present study and subjective nature of the responses (self-reported) may portray an overestimate of the true prevalence than a physician diagnosed allergy. In vitro IgE determination or in vivo skin prick test was not performed to identify the cause of allergies in these students.

## 5. Conclusion

Allergic conjunctivitis and allergic dermatitis were the frequent allergies reported. The study findings support the recent evidences of increasing prevalence of allergic diseases in the Middle Eastern region. The observations of the study highlight the need for future studies with larger sample to determine the true prevalence. Adequate preventive strategies such as creating awareness of allergic disease prevalence and its risk factors and treatment options can bring down mortality, morbidity, and disability caused by this public health problem.

## Figures and Tables

**Table 1:** Sociodemographic characteristics of university students.

Characteristic	Item	Number	Percentage
Gender	Male	85	33.3
	Female	170	66.7
Age	Mean ± standard deviation	20.1 ± 2.58 years	
Course	Medicine	150	58.8
	Dentistry	59	23.1
	Pharmacy	10	3.9
	Physical therapy	36	14.1
Length of stay	<1 year	39	15.3
	1–5 years	61	23.9
	5–10 years	37	14.5
	10–15 years	23	9.0
	≥15 years	95	37.3
Nationality	India	37	14.5
	Pakistan	38	14.9
	Other Asians	12	4.7
	Africa	36	14.1
	United Arab Emirates	31	12.2
	Other Middle Easterns	54	21.2
	Europe	10	3.9
	Others	37	14.5

**Table 2:** Gender-based distribution of allergic disorders among university students.

Allergies	Male	Female	Total	*P* value
Allergic conjunctivitis	25 (24%)	79 (76%)	104	<0.05
Allergic dermatitis	21 (23.5%)	66 (76.5%)	89	NS
Eczema	12 (31.5%)	26 (68.5%)	38	NS
Dust allergies	18 (31%)	40 (69%)	58	NS
Food allergies	6 (25%)	18 (75%)	24	NS
Drug allergies	6 (33.3%)	12 (66.7%)	18	NS
Pollen allergies	4 (30.7%)	9 (69.3%)	13	NS

## Appendix

### Questionnaire

Gender:

Age:

Nationality:

Length of stay in UAE

(1) In the past 12 months, have you had a problem with sneezing, or a runny, or a blocked nose when you did not have a cold or flu?
  Yes/No
(2) In the past 12 months, have you ever had itchy-watery eyes?
  Yes/No
(3) In the past 12 months, have you ever had an itchy rash?
  Yes/No
(4) In the past 12 months, have you ever had eczema?
  Yes/No
(5) In which of the past 12 months do you have these problems?
  January/February/March/April/ May/June/July/August/September/October/November/December
(6) Do any you have allergies to any of the following? Dust/Pollen/Food/ Drug/others
  If yes, please specify –––––––––––
(7) Do any of your family members have any of the following allergies?
*Allergy*
Dermatitis
  *  *Yes No
  *  *If yes,
    Mother  Father  Sister Brother
Allergic rhinitis
  * *Yes No
  * *If yes,
    Mother  Father  Sister Brother
Allergic conjunctivitis
  * *Yes No
  If yes,
    Mother  Father  Sister Brother
(8) Has your allergies interfered with any of the following?
  College attendance
    Yes No
  Exam performance
    Yes No
  Daily Activities
    * *Yes No
  Attending social gathering
    Yes No
  Extracurricular activities (sports, hobbies, exercise…)
    Yes No
